# The National Pension Fund

**Published:** 1887-10-01

**Authors:** 


### The National Pension Fund.

It will save a good deal of trouble if nurses and other hospital officials who propose joining the National Pension Fund will send their names and other particulars to *the Secretary of The Hospitals Association*, Norfolk House, Norfolk Street, W.C., and not to the Editor of The Hospital. Names continue to arrive in considerable numbers, but if those interested desire the Fund to be started before the end of the Jubilee year they must influence and interest all their friends immediately.

Readers will please note that in sending in their names, nurses and other hospital officials are not committing themselves to join any particular Fund, but simply stating their willingness to join "a Pension Fund if the rules and regulations are such as they approve."

---

Over nine hundred names have now been sent in for the National Pension Fund. All hospital officials and nurses should make a point of attending the meeting of The Hospitals Association on the 12th October, under the presidency of Sir Andrew Clark. A paper will then be read, dealing thoroughly with the subject, and it ought to be possible then and there to make a definite commencement of the scheme. Country officials and nurses coming up for the meeting will be cordially welcomed.

---

From a Nursing Institution.

It gives me much pleasure to say how highly we think of The Hospital; we have quite got to look forward to receiving it weekly. I wish to enter my name on the list of those who approve of the Nurses' Pension Fund, and when it is really settled, should like to become a member. Most of the nurses here are in favour of the fund, and I feel sure many of them would join. Wishing you all success in your good paper and hoping to hear soon of a decided settlement. L. C.

